# Adipose‐derived mesenchymal stromal cells modulate experimental autoimmune arthritis by inducing an early regulatory innate cell signature

**DOI:** 10.1002/iid3.106

**Published:** 2016-04-04

**Authors:** Mercedes Lopez‐Santalla, Ramon Menta, Pablo Mancheño‐Corvo, Juan Lopez‐Belmonte, Olga DelaRosa, Juan A. Bueren, Wilfried Dalemans, Eleuterio Lombardo, Marina I. Garin

**Affiliations:** ^1^ Hematopoietic Innovative Therapies DivisionCentro de Investigaciones Energéticas, Medioambientales y Tecnológicas (CIEMAT) and Centro de Investigación Biomédica en Red de Enfermedades Raras (CIBER‐ER)MadridSpain; ^2^ Advanced Therapy UnitCentro de Investigaciones Energéticas, Medioambientales y Tecnológicas (CIEMAT) and Instituto de Investigación Sanitaria Fundación Jiménez Díaz (IIS‐FJD, UAM)MadridSpain; ^3^ TiGenix SAUMadridSpain; ^4^ FARMACROSAlbaceteSpain; ^5^ TiGenix NVLeuvenBelgium

**Keywords:** Adipose‐derived mesenchymal stem cells, arthritis, IL10^+^F4/80^+^ macrophages, Ly6C^+^ monocytes

## Abstract

Modulation of innate immune responses in rheumatoid arthritis and other immune‐mediated disorders is of critical importance in the clinic since a growing body of information has shown the key contribution of dysregulated innate responses in the progression of the disease. Mesenchymal stromal cells (MSCs) are the focus of intensive efforts worldwide due to their key role in tissue regeneration and modulation of inflammation. In this study, we define innate immune responses occurring during the early course of treatment with a single dose of expanded adipose‐derived MSCs (eASCs) in established collagen‐induced arthritis. eASCs delay the progression of the disease during the early phase of the disease. This is accompanied by a transient induction of Ly6C^+^ monocytes that differentiate into IL10^+^F4/80^+^ cells in arthritic mice. Strikingly, the induced IL10^+^F4/80^+^ myeloid cells preferentially accumulated in the draining lymph nodes. This effect was accompanied with a concomitant declining of their frequencies in the spleens. Our results show that eASCs attenuate the arthritic process by inducing an early innate cell signature that involves a transient induction of Ly6C^+^ monocytes in periphery that differentiate into IL10^+^F4/80^+^ macrophages. Our findings demonstrate that early regulatory innate cell responses, involving the monocyte compartment, are targeted by the eASCs during the onset of collagen‐induced inflammation.

## Introduction

Rheumatoid arthritis (RA) is an autoimmune disease of unknown etiology that is characterized by synovial hyperplasia and cartilage/bone destruction with systemic comorbidities. Accumulating data show that CD4 T cells, especially IL‐17‐producing T helper (Th17), and neutrophils play a significant role during the chronic inflammation 1, 2. In recent years, myeloid‐derived suppressor cells (MDSCs) have also attracted considerable attention by their increase in RA patients 3, 4, 5 and experimental models of arthritis 4, 5, 6, 7, 8. In mice, they are defined as Gr1^+^ CD11b^+^ cells with a suppressive effector function. Based on the expression of Ly6G and Ly6C molecules, two subsets of MDSCs have been described, i.e., the granulocytic MDSCs defined as Ly6G^+^Ly6C^low^ CD11b^+^ cells and the monocytic MDSCs defined as Ly6G^−^Ly6C^hi^CD11b^+^ cells 9, 10. At present, disagreements exist on the role played by the MDSCs in RA 3, 4, 5, 6, 7, 8. Their anti‐inflammatory function in RA has been claimed by several groups 3, 6, 7, 8, while other reports have shown their proinflammatory role during the progression of experimental arthritis as well as in patients with RA 4, 5.

Despite major progress in the understanding of pathogenesis of RA, strong unmet medical need remains 11. New approaches are, therefore, necessary and mesenchymal stem cells (MSCs) could represent a valuable therapeutic strategy for RA 12, 13, 14, 15. The use of MSCs in the clinical field has gathered tremendous momentum over the last decade, advanced by varying levels of success in clinical trials 13, 16, 17, 18, 19 and by the progress in our understanding of their mechanisms of action 20, 21, 22. Preclinical and clinical studies have demonstrated that MSCs attenuate inflammatory response by induction of regulatory T cells 13, 23, 24, 25, secretion of molecules with anti‐inflammatory effects 26, inhibition of dendritic cell maturation 27, and generation of macrophages with regulatory phenotype 28, 29, 30, 31, 32, 33, among others. Number of studies have demonstrated that MSCs, either in vitro and in vivo, can induce MDSCs 29, 30, 31, 32, 33, 34 and these populations are responsible for the beneficial effects of the MSCs in modulating the inflammation 29, 30, 32, 33, 34, 35.

The majority of the in vivo studies with eASCs for preventing collagen‐induced arthritis used multiple doses of eASCs before the onset of the disease 36, 37, 38. Several studies have demonstrated that multiple doses of eASCs can have a sustained beneficial effect in a therapeutic protocol 23, 37. The sustained effect observed when multiple doses of eASCs are used might be the result of a very complex response which may not be easily explained by direct interaction with the eASCs. We have recently demonstrated that a single dose of eASCs during the onset of the disease significantly decrease the severity of the arthritis and this was accompanied by the induction of different subsets of regulatory T cells and IL10‐producing Th17 cells 25. In this context, we hypothesized whether cell therapy with eASCs also induces early innate responses that would contribute to the reestablishment of the regulatory/inflammatory balance during the ongoing inflammation. Our findings demonstrate that an early regulatory innate response, involving the monocyte compartment, is induced soon after the infusion the eASCs which may lead to an effective modulation of the ongoing inflammation.

## Results

### eASC treatment delayed the progression of established disease in experimental arthritis

**Figure 1:**
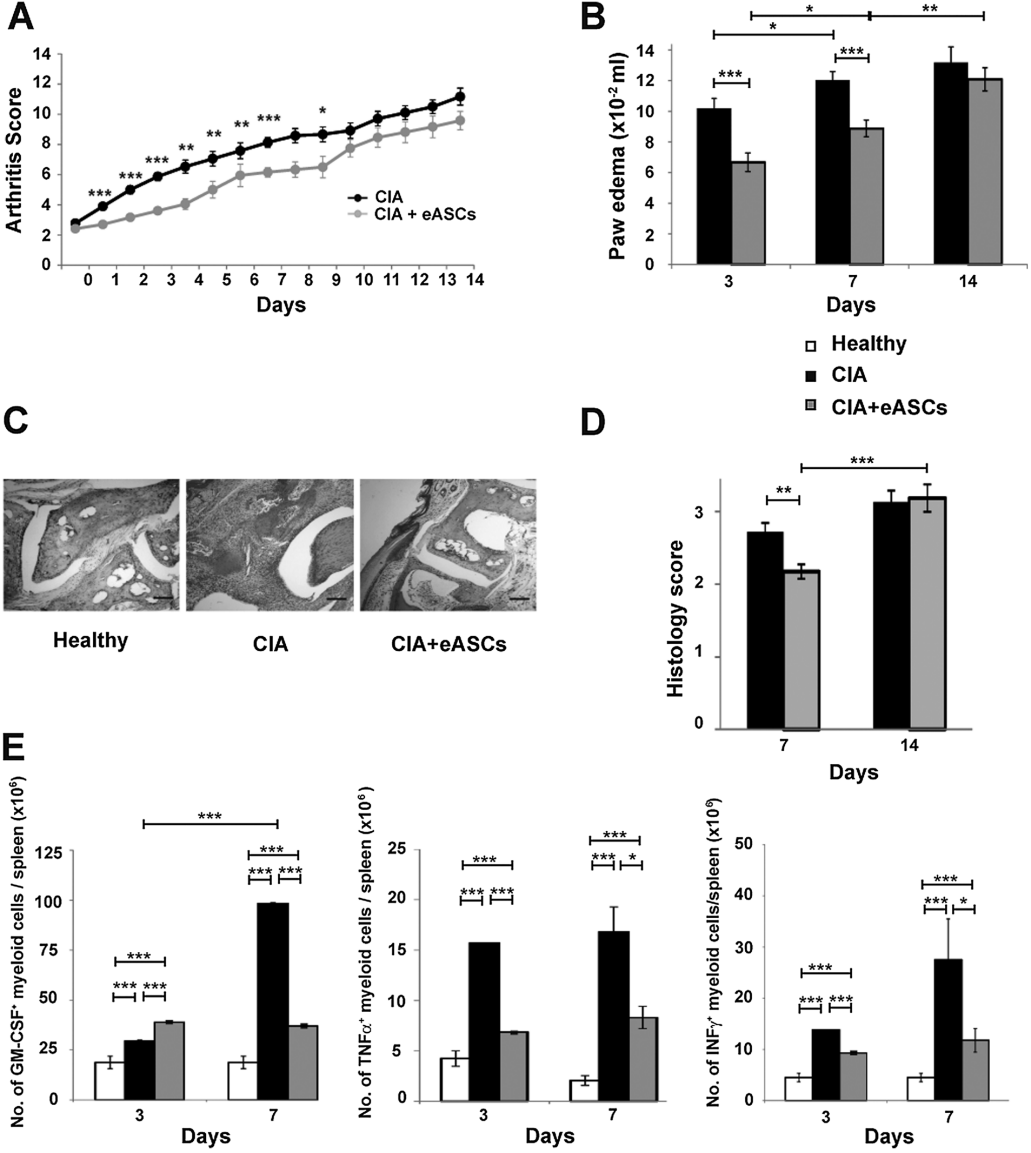
Arthritis status of mice measured by arthritis score, paw edema, histology, and number of inflammatory myeloid cells. Arthritis score (A), based on the number of swollen paws (front and hind), was evaluated daily for 14 days in the CIA (*n* = 49) and eASC‐treated CIA (*n* = 67) mice. (B) Paw edema is measured at day 3, 7, and 14 by a plethysmometer in CIA (*n* = 34) and eASC‐treated CIA (*n* = 52) mice. (C) Representative sections showing examples of paw swelling in healthy, CIA, and eASC‐treated CIA mice. Scale bars, 200 μm. Original magnification, ×40. (D) Histology score based on synovitis and cell infiltration evaluated at days 7 and 14 in the CIA (*n* = 12) and eASC‐treated CIA (*n* = 20) mice. (E) Quantification of GM‐CSF, TNFα, and INFγ inflammatory myeloid cells in spleen by flow cytometry. Cells were activated with PMA and ionomycin for 4 h in the presence of GolgiStop and GolgiPlug. After incubation, cells were harvested and stained on their surface. For intracellular staining, cells were fixed permeabilized and stained. Percentages of GM‐CSF, TNFα, and INFγ inflammatory myeloid cells in the spleen at day 3 and 7 of healthy, CIA, and eASC‐treated CIA mice were shown. Healthy *n* = 24, CIA *n* = 25, and eASC‐treated CIA *n* = 42 for GM‐CSF and TNFα myeloid cells and healthy, *n* = 24; CIA, *n* = 13, and eASC‐treated CIA, *n* = 22 for INFγ myeloid cells. Data are presented as mean and the standard error of the mean of the arthritis score, paw edema, histology score, and number of inflammatory myeloid cells. Significance was analyzed by the Mann–Whitney test and represented by *P < 0.05, **P < 0.01 and ***P < 0.001. Results represent four experiments.

To study early innate cell responses induced by administration of eASCs, we first evaluated the progression of inflammation in a collagen‐induced mice model (CIA) treated or not with eASCs. A single dose of 1 × 10^6^ eASCs/mouse was administered intravenously (day 0) at the onset of the detectable disease signs (arthritis score between 2 and 4). As shown in Figure 1A, from day 1, 24 h after the infusion of the eASCs, there was a statistically significant delay in the progression of the disease in eASC‐treated CIA mice compared to the CIA mice. From day 9 onwards, the differences observed were lost. The fact that the effect of the eASCs was transient was somewhat expected as previous studies have demonstrated that multiple doses of eASCs can have a sustained beneficial effect when used in a therapeutic protocol 23, 37. According to this kinetic, the analyses of cell responses were conducted from day 0 to 7 when the modulatory effects of the eASCs were clearly distinguished. The beneficial effects of eASCs were further confirmed by a reduction in paw edema at days 3 and 7 in the eASC‐treated CIA mice (Fig. 1B) and by histology analysis (Fig. 1C and D) of the whole ankle joints. At day 7, the modulation of inflammation was also confirmed by analysis of inflammatory myeloid populations in the spleen. As shown in Figure 1E, the frequencies of myeloid cells expressing GM‐CSF, TNFα, and INFγ were significantly reduced in eASC‐treated CIA mice compared to CIA mice. The GM‐CSF‐expressing myeloid cells also co‐expressed INFγ and TNFα although to a lesser extent than the GM‐CSF cytokine (data not shown).

### eASC treatment induced a transient increase in monocytes in peripheral blood of arthritic mice

**Figure 2:**
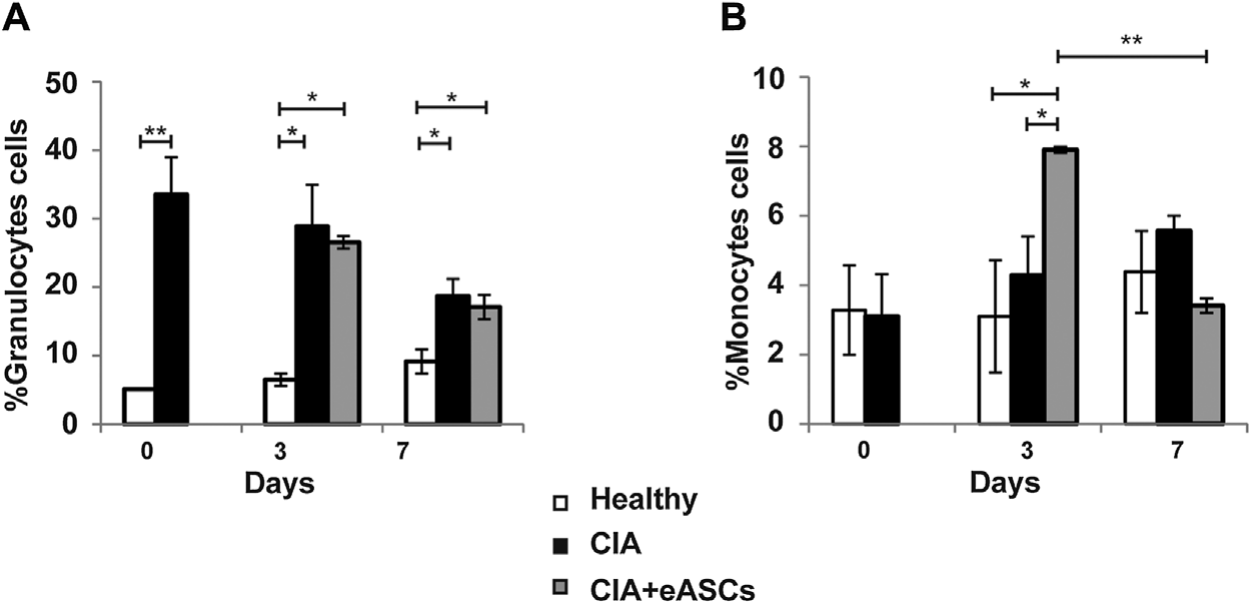
Hematological analysis of peripheral blood samples. Peripheral blood was analyzed by an automated blood cell‐counter. (A) Percentage of granulocytes cells and (B) percentage of monocytes cells of healthy, CIA, and eASC‐treated CIA mice at day 0, 3, and 7. Data are represented by the mean and the standard error of the mean. *P < 0.05 and **P < 0.01 represent the significance analysed by the Mann‐Whitney test (healthy, *n* = 9; CIA, *n* = 14; and eASC‐treated CIA, *n* = 12). Data correspond to one representative out of four experiments.

The progression of inflammation was monitored by hematological analysis of peripheral blood (PB) samples. As expected, mice with CIA had a marked granulocytosis in circulation. Treatment with eASCs had no influence in the granulocytes (Fig. 2A). Surprisingly, at day 3, there was an increase in the percentage of monocytes in the eASC‐treated CIA mice compared to CIA mice. At day 7, the percentage of monocytes in eASC‐treated CIA mice did not differ from those of CIA and healthy mice (Fig. 2B). These results suggested that, as a consequence of the infusion of the eASCs in CIA mice, there was a transient increase in monocytes in circulation. This observation indicated that early innate responses, within the monocyte compartment, were actively participating in the cell responses induced by the eASCs.

### The transient increase of monocytes in PB was due to a rapid induction of Gr1^+^CD11b^+^ myeloid cells

**Figure 3:**
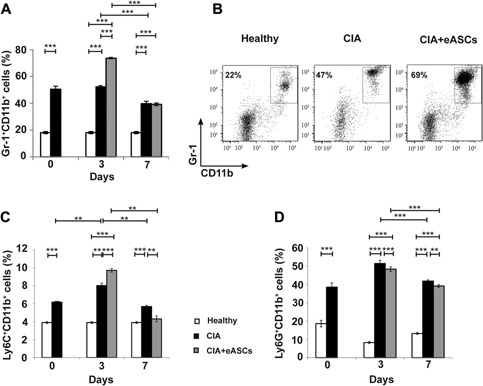
Quantification of Gr‐1^+^CD11^+^, Ly6C^+^CD11b^+^, and Ly6G^+^CD11b^+^ myeloid cell populations in peripheral blood by flow cytometry. Cells were stained on their surface with anti‐CD11b, anti‐Gr‐1, anti‐Ly6C, and anti‐Ly6G monoclonal antibodies. (A) Percentage of Gr‐1^+^CD11b^+^ myeloid cells in peripheral blood at day 0, 3, and 7, (B) representative dot plot of Gr‐1^+^CD11b^+^ myeloid cell population in peripheral blood at day 3, (C) percentage of Ly6C^+^CD11b^+^ myeloid cells at day 0, 3, and 7 in peripheral blood, and (D) percentage of Ly6G^+^CD11b^+^ myeloid cells at day 0, 3, and 7 in peripheral blood of healthy, CIA, and eASC‐treated CIA mice. Data are represented by the mean and the standard error of the mean. **P < 0.01 and ***P < 0.001 represent the significance analyzed by the Mann–Whitney test (healthy, *n* = 20; CIA, *n* = 19; and eASC‐treated CIA, *n* = 23). Results represent four experiments.

Peripheral blood samples were analyzed by flow cytometry in order to identify the myeloid populations that were induced by the eASCs. Gr1^+^CD11b^+^ myeloid cells were increased in CIA mice when compared to healthy mice. The infusion of eASCs led to a transient induction of Gr1^+^CD11b^+^ myeloid cells in the eASC‐treated CIA mice compared to CIA mice. These differences were not observed at day 7 (Fig. 3A and B).

The granulocyte receptor‐1 (Gr1, detected by the clone RB6‐8C5 monoclonal antibody) was initially believed to be expressed only by mature granulocytes. Later on, studies have demonstrated that the RB6‐8C5 antibody reacts with the structurally glycophosphatidylinositol (GPI)‐anchored proteins; Ly‐6G (21–25 kDa), expressed by granulocytes, and Ly‐6C (14–16 kDa), expressed by several subsets of monocytes/macrophages as well as dendritic cells and lymphocytes. To further define the subsets of myeloid populations that were induced by the eASCs, PB samples were labeled with specific monoclonal antibodies to Ly6C (clone 1G7.G10) and Ly6G (clone 1A8) antigens. As shown in Figure 3C and Supplementary Figure S1A, Ly6C^+^CD11b^+^ cells were increased in CIA mice compared to healthy mice. Strikingly, upon treatment with eASCs, Ly6C^+^CD11b^+^ cells were transiently increased at day 3 compared to CIA mice. At day 7, the levels of Ly6C^+^CD11b^+^ cells in circulation decreased in both CIA mice, treated or not with eASCs (Figure 3C and Supplementary Figure S1A).

On the other hand, the frequencies of Ly6G^+^CD11b^+^ cells were significantly increased in both CIA and eASC‐treated CIA mice, respectively to healthy mice (Fig. 3D and Supplementary Figure S1B). Altogether, these data suggested that the transient increase of Gr1^+^CD11b^+^ myeloid cells observed in the eASC‐treated CIA mice was due to the contribution of the Ly6C^+^ CD11b^+^ myeloid cells and not to the Ly6G^+^CD11b^+^ cells.

### eASCs primed Ly6C^+^ monocytes to a regulatory IL10^+^F4/80^+^ phenotype favoring their trafficking to dLNs

**Figure 4:**
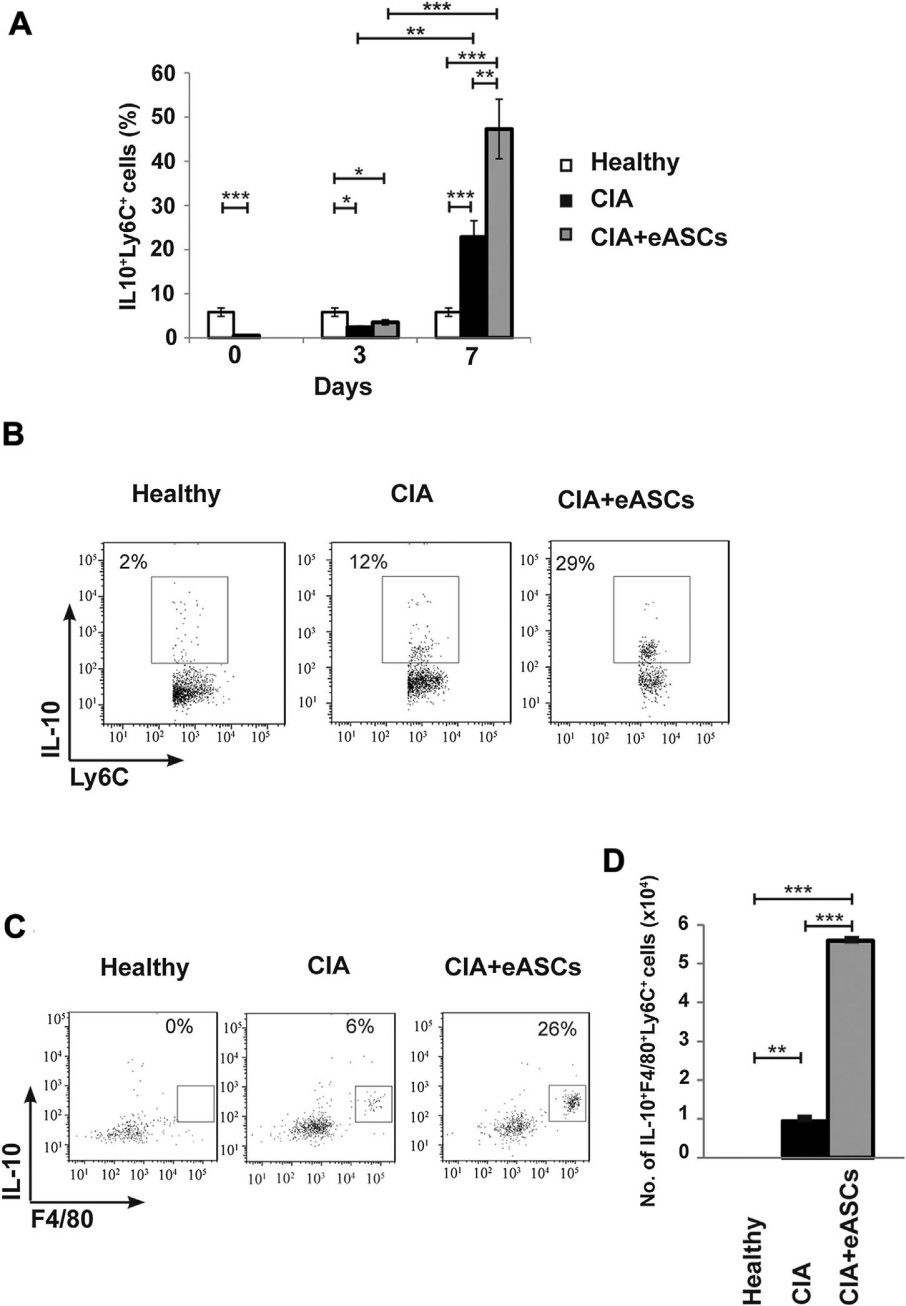
Quantification of IL‐10^+^Ly6C^+^ and IL10^+^F4/80^+^ within Ly6C^+^ myeloid cell populations in peripheral blood by flow cytometry. Cells were activated with PMA and ionomycin for 4 h in the presence of GolgiStop and GolgiPlug. After incubation, cells were harvested and stained on their surface with anti‐Ly6C and anti‐F4/80 monoclonal antibodies. For intracellular staining, cells were fixed, permeabilized, and stained with anti‐IL‐10 monoclonal antibody. (A) Percentage of IL‐10^+^Ly6C^+^ myeloid cell populations in peripheral blood at day 0, 3, and 7, (B) representative plot of IL‐10^+^Ly6C^+^ myeloid cells at day 7 in peripheral blood, (C) representative plot of IL10^+^F4/80^+^ myeloid cell within the Ly6C^+^ myeloid cells at day 7 in peripheral blood, and (D) number of IL‐10^+^F4/80^+^Ly6C^+^ myeloid cells in peripheral blood at day 7 of healthy, CIA, and eASC‐treated CIA mice. Data are represented by the mean and the standard error of the mean. *P < 0.05, **P < 0.01, and ***P < 0.001 represent the significance analyzed by the Mann–Whitney test (healthy, *n* = 12; CIA, *n* = 13; and eASC‐treated CIA, *n* = 14). Results represent four experiments.

Ly6C^+^ monocytes play multiple roles upon tissue recruitment and the differentiation and effector functions of these cells can be shaped according to the environment, both at steady state and during inflammation 39, 40. To explore the possibility as to whether the eASCs can prime Ly6C^+^ monocytes for regulatory function, we analyzed the expression of IL10 within the Ly6C^+^ monocytes in PB. Interestingly, at day 7, eASC‐treated CIA mice had a significant increase in the proportion of Ly6C^+^CD11b^+^ myeloid cells co‐expressing IL10 compared to CIA mice. In CIA mice, these populations were also induced when compared to healthy mice, most likely as a consequence of the ongoing inflammation (Fig. 4A and B). This observation may suggest that the induction of the IL10^+^Ly6C^+^CD11b^+^ myeloid cells occurred in CIA as a consequence of the disease and the treatment with the eASCs amplified this regulatory innate response induced by the ongoing inflammation.

**Figure 5:**
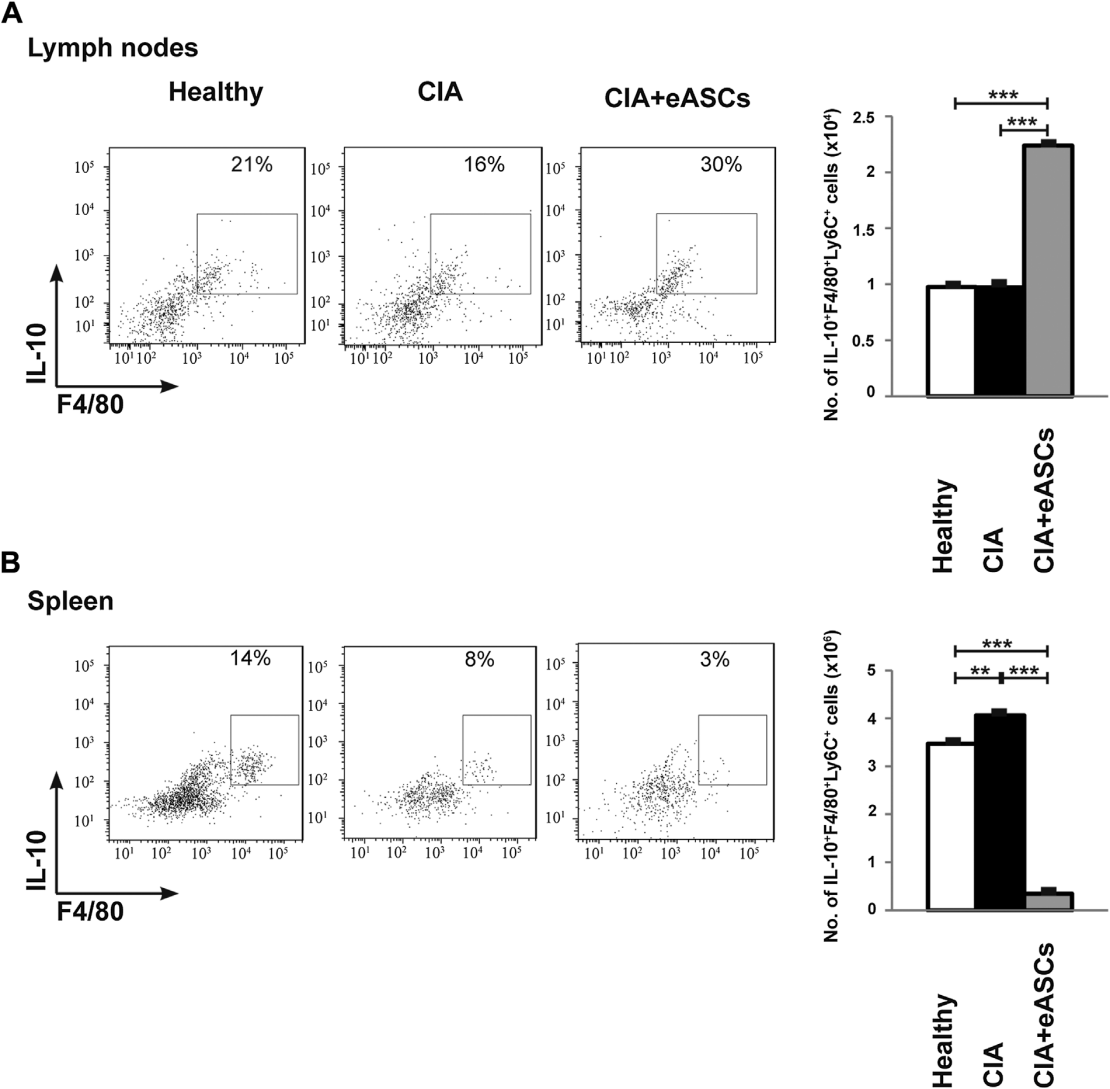
Quantification of IL10^+^F4/80^+^Ly6C^+^ myeloid cell populations in draining lymph nodes and spleen by flow cytometry. Cells were activated with PMA and ionomycin for 4 h in the presence of GolgiStop and GolgiPlug. After incubation, cells were harvested and stained on their surface with anti‐Ly6C and anti‐F4/80 monoclonal antibodies. For intracellular staining, cells were fixed, permeabilized, and stained with anti‐IL‐10 monoclonal antibody. (A) Representative plot and number of IL‐10^+^F4/80^+^Ly6C^+^ myeloid cell populations in dLNs at day 7 and (B) representative plot and number of IL‐10^+^F4/80^+^ Ly6C^+^ myeloid cell populations myeloid cells in spleen at day 7 of healthy, CIA, and eASC‐treated CIA mice. Data are represented by the mean and the standard error of the mean. **P < 0.01 and ***P < 0.001 represent the significance analyzed by the Mann–Whitney test (healthy, *n* = 5; CIA, *n* = 7; and eASC‐treated CIA, *n* = 15). Results represent four experiments.

At day 7, there was a significant increase in the number of IL10^+^F4/80^+^ cells within the Ly6C^+^ myeloid cells in eASC‐treated CIA mice, compared to CIA mice, indicating that these populations actually differentiated into regulatory macrophages in circulation (Fig. 4C and D). This population was also greatly increased in the dLNs of CIA mice treated with eASCs. In sharp contrast, the number of IL10^+^F4/80^+^ cells within the Ly6C^+^ myeloid cells was dramatically reduced in the spleen of eASC‐treated CIA mice compared to CIA and healthy mice (Fig. 5). These findings may indicate that the eASC treatment induced the skewing of Ly6C^+^ monocytes to a regulatory phenotype and their accumulation to the dLNs where the inflammation was taking place.

## Discussion

Here, we show that cell therapy with eASCs, among other effects, modulate the inflammatory innate cell responses with a concomitant delay in the progression of the arthritis. Modulation of innate immune responses in RA is of critical importance in the clinic since a growing body of evidence has shown the key contribution of dysregulated innate responses during the progression of the disease 1, 41. The fact that the effect of the eASCs was transient was somewhat expected. The majority of the in vivo studies with eASCs for preventing collagen‐induced arthritis used multiple doses of MSCs before the onset of the disease 36, 37, 38. Gonzalez MA and collaborators demonstrated that three doses of eASCs can have a sustained beneficial effect when used in a therapeutic protocol 23. In the present study, we have used a single dose of eASCs for treating arthritic mice when the score of arthritis was between 2 and 4. This experimental strategy allowed us to dissect in great detail, the cell responses induced upon the infusion of the eASCs in an inflammatory environment. The sustained effect observed when multiple doses of eASCs are used for treating arthritic mice might be the result of a very complex response which may not be easily explained by direct interaction with the eASCs.

In our CIA experiments, arthritic mice rapidly developed a strong innate response soon after the challenge with the collagen II antigen. In the periphery, mice with CIA had a marked granulocytosis. The inflammatory nature of the granulocytes was confirmed by the expression of GM‐CSF, TNFα, and INFγ cytokines in myeloid cells. The modulatory effects of the eASCs during the onset of the disease had a major impact on these populations as CIA mice treated with eASCs had reduced number of inflammatory myeloid cells (Fig. 1E). This is a very interesting effect of the eASCs since the majority of therapeutic approaches mediate their beneficial action through cytokine blockade such as TNFα, IL6, IL1, and GM‐CSF 2, 11, 42, 43, 44, 45 rather than targeting the inflammatory cells themselves. Currently, ongoing clinical trials using monoclonal antibodies against the GM‐CSF protein or GM‐CSF receptor α have shown significant responses in RA patients 43, 44, 45. This would imply that the cell therapy with eASCs can target the underlying cause of the disease and not the inflammation itself.

Although compiling data have demonstrated that mouse and human eASCs differ in some of their mechanisms of immunomodulation, studies in preclinical models of arthritis using human eASCs allow to dissect pathways shared by the human and mouse systems. Moreover, we have recently shown that eASCs are short‐lived after in vivo administration 46, even when used in a syngeneic setting 47, indicating that eASCs, regardless the MHC context, can prime host immune cells through an array of not fully understood specific molecular mechanism, which, in turn, adopt a regulatory phenotype.

Of interest in this study, the hematological analysis revealed a transient increase in the frequencies of monocytes in circulation in CIA mice treated with eASCs (Fig. 2). By flow cytometry, the phenotype of these cells corresponded to Gr1^+^CD11b^+^ myeloid cells (Fig. 3). Our data are in accordance with previous in vitro and in vivo studies where it has been shown that MSCs favored the induction of Gr1^+^CD11b^+^ myeloid cells with a regulatory phenotype 29, 30, 33, 35, 48. These cells have been described as heterogeneous populations of myeloid cells with immunoregulatory properties also known as myeloid derived suppressor cells (MDSCs) 9, 10. It is well known that MDSCs accumulate in RA patients 3, 4, 5 and in experimental inflammation including CIA 4, 5, 6.

Gr1^+^CD11b^+^ regulatory myeloid cells are able to modulate adaptive immune responses through induction of different populations of regulatory T cells in vitro 48, 49. Recently, we have demonstrated that eASCs modulate ongoing immune responses by promoting an early adaptive regulatory T cell signature. In that study, we found increased levels of regulatory T cells and plasticity of effector Th17 cells toward an IL10‐driven anti‐inflammatory response. Ultimately, these cell responses led to restoring the regulatory/inflammatory balance following the onset of the disease 25. These observations allowed us to hypothesize whether the eASCs orchestrate the adaptive regulatory T cell responses through a mechanism involving innate responses.

The role of MDSCs during the development of RA is controversial. Gr1^+^CD11b^+^ cells are composed by two different subsets of cells based on the expression of Ly6C and Ly6G molecules. Our data showed that the transient increase in the Gr1^+^CD11b^+^ cells in the eASC‐treated CIA mice was mainly due to the contribution of the Ly6C^+^CD11b^+^ cells. In eASC‐treated CIA mice, the expression of IL10 within Ly6C^+^CD11b^+^ cells was higher than in CIA mice. Furthermore, one week after the infusion of the eASCs, the majority of IL10^+^Ly6C^+^CD11b^+^ cells expressed the F4/80 macrophage marker. These data are in agreement with Nemeth and collaborators where, in a model of sepsis, increase frequencies of Gr1^+^F4/80^+^ myeloid cells expressing IL10 upon treatment with MSCs were observed. Neutralisation of IL10 or the IL10 receptor clearly reduced the protective effects of the MSCs 29. Lee et al. 33, using a mouse model of experimental autoimmune uveitis, observed that intravenous infusion of MSCs led to an accumulation of Ly6C^+^CD11b^+^ cells in draining lymph nodes and that depletion of the Gr‐1^+^ cells, by neutralizing antibody against Gr‐1, abrogated the effects of the MSCs. In line with these observations, in a model of solid organ transplantation, Obermajer et al., demonstrated the induction of F4/80^+^Gr1^+^CD11b^+^ MDSCs by the MSCs. Ultimately, the MDSCs induced the plasticity of the Th17 cells toward ex‐IL17 Foxp3^+^ regulatory T cells 32. In support of this link, the increase of regulatory myeloid cells in the dLNs found in this study is accompanied by the increase of regulatory T cells as described in our previous report 25.

Different reports endorsed that the immunomodulatory capacity of MSCs takes place both by direct cell‐to‐cell contact and by means of soluble factors. Gonzalez‐Rey et al. 24 proposed that hASC–monocyte cocultures produced high amounts of IL10 through a cell to cell contact‐dependent mechanism. It has been demonstrated that bone marrow MSCs (BM‐MSCs) induce monocytes and dendritic cells in a cell contact‐dependent manner to secrete high IL10 amounts 50. Nemeth et al. proposed that both mechanisms, both direct contact and secreted factors, induced the secretion of IL10 by LPS‐stimulated macrophages in co‐culture experiments with MSCs; although the levels are significantly higher when the LPS‐macrophages were in direct contact with MSCs than when they were cultured in transwell plates without a direct contact with the MSCs or exposed to MSC‐conditioned medium 29.

Other groups claimed that secreted factors produced by the MSCs are responsible for the skewing of monocytes toward an anti‐inflammatory function. In this sense, 33, 51, 52 Yen et al. 53 suggested that the expansion of Gr‐1^+^CD11b^+^ MDSCs occurs through the secretion of hepatocyte growth factor (HGF) secreted by the MSCs that involves c‐Met (the HGF receptor) and downstream phosphorylation of STAT3, a key factor that favors the expansion of MDSC. Melief et al. 31 proposed that the factor implicated in the skewing of the monocytes to a regulatory phenotype is via IL6. In line with these observations, Campioni et al. 54 proposed that the production of soluble HLA‐G by the MSCs is a key molecule mediating their interaction with monocytes.

Interestingly, the IL10^+^F4/80^+^Ly6C^+^ myeloid cells accumulated in the PB and dLNs whereas they were reduced in the spleens in eASC‐treated CIA mice. These data suggested that eASCs could favor their migration from spleen to dLNs which could be an additional effect leading to a delayed progression of CIA. In a recent study, Lee et al. have shown that MSCs modulate immune responses by a mechanism that involves the recruitment of the Ly6C^+^Ly6G^−^CD11b^+^ MDSCs to the dLNs in a CCL2‐dependent manner, thus, reducing the number of Th1 and Th17 cells in periphery 33. At present, the reactive dLNs are the object of growing attention when monitoring patients with chronic inflammatory diseases being a relevant tool to investigate immunological changes in the early phases and during the progression of arthritis 55.

In summary, our data demonstrate that eASCs modulate ongoing inflammatory innate responses in vivo by a transient increase of Ly6C^+^ monocytes in PB. By day 7, increased frequencies of IL10^+^F4/80^+^Ly6C^+^ regulatory monocytes were found in periphery in eASC‐treated CIA mice (Supplementary Figure S2). The presence of the eASCs during the onset of inflammation may have an impact on their trafficking as shown by their preferential accumulation in the dLNs. These results open new avenues for treating RA and other inflammatory‐mediated diseases where early innate responses could favor sustained adaptive regulatory T cell responses at the inflamed tissues.

## Materials and Methods

### Mice

DBA/1 male mice of 6–8 weeks of age were obtained from Janvier SAS, France. All experiments were performed in accordance with the corresponding regulations regarding experimental animal welfare (RD 223/1988 and Directive 2010/63/EU protocols) and approved by the Institutional Animal Care and Use Committee at the University of Albacete, Spain.

### Generation of human expanded adipose‐derived stromal cells

Human samples were obtained after informed consent as approved by the Spanish Ethics Committee of reference for the site of tissue procurement (Clínica de la Luz Hospital, Madrid, Spain). Human adipose tissue aspirates from healthy donors were processed as described elsewhere 25. All the eASCs used fulfilled the release criteria of identity, purity, and potency needed for their clinical use.

### Induction and evaluation of collagen‐induced arthritis (CIA) and treatment with eASCs

Mice were injected subcutaneously in the tail with an emulsion of Chicken Collagen Type II (1 mg/mL, Chondrex, Redmond, CA). A second injection was administered 21 days after the first injection. Clinical signs of arthritis were evaluated daily after the second immunization to determine clinical evidence of arthritis as described 25.

Treatment with one million eASCs was administered intravenously (tail vein) (Grifols, Madrid, Spain) when an arthritis index score of 2–4 was attained 36, 46, 56. The Arthritis Index Score was conducted until the end of the study according to Ref. 25.

At days 0, 3, and 7 cells were isolated from PB. Complete blood counts were obtained using an automated blood cell‐counter (Abacus, Diatron, Budapest, Hungary). At days 3 and 7, mice were culled and mononuclear cells were isolated from spleen and dLNs (inguinal and popliteal) using a cell strainer. A Neubauer chamber was used to determine the number of cells.

### Histology and paw edema analysis

At days 7 and 14, paws were fixed in neutral buffered formalin; 3–4 sections were obtained from each paw and stained with hematoxylin and eosin. The degree of inflammation in each section was assessed according to Ref. 25. Paw edema was assessed daily as the volume of both hind and front paws by the use of a plethysmometer.

### Flow cytometry analysis

Isolated mononuclear cells from PB, spleen and dLNs were surface stained with antibodies directed against mouse CD45PerCP (30F11.1), Ly6C‐FITC (1G7.G10), and Ly6G‐PerCPVio770 (1A8) from Miltenyi Biotech (Bergisch Gladbach, Germany), F4/80‐PECy5 (BM8) from eBiosciences (San Diego, CA), streptavidin‐BV650 from Biolegend (San Diego, CA), and CD11b‐biotin (M1/70), Gr1‐biotin (RB6‐8C5), Ly6G‐BV421 (1A8), and isotype‐matched control IgG from Becton Dickinson Pharmingen (Franklin Lakes, NJ). Cells were collected on a BD LSR Fortessa flow cytometer (Becton Dickinson). At least 10,000 events were acquired. Data were analyzed using FlowJo software (Ashland, OR).

### Cytokine analysis

For intracellular analysis of cytokine expression, mononuclear cells were stimulated in vitro with 5 ng/mL phorbol myristate acetate (PMA; Sigma–Aldrich) and 500 ng/mL ionomycin (Sigma–Aldrich) for 4 h. GolgiStop and GolgiPlug (BD Pharmingen) were added after 1 h. Cells were fixed and stained according to manufacturer instructions (Cytokine BD kit, BD Pharmingen). For intracellular staining, GM‐CSF‐FITC (MP1‐22E9), TNFα (MP6‐XT22), and INFγ (XMG1.2) from eBiosciences and IL10‐FITC (JES5‐16E3) from BD Pharmingen were used.

### Statistical analysis

Data are presented as the mean and the standard error of the mean. Normal distribution was analysed by the Shapiro–Wilks test. Non‐parametric techniques (Mann–Whitney *U*‐test) were used. Analyses were performed using the software Stata 11 (StataCorp, College Station, TX).

## Conflicts of Interest

None declared.

## Supporting information

Additional supporting information may be found in the online version of this article at the publisher's web‐site.

**Figure S1**. Quantification of Ly6C^+^CD11b^+^ and Ly6G^+^CD11b^+^ myeloid cell populations in peripheral blood by flow cytometry.

**Figure S2**. Schematic model for early innate responses induced by the expanded adipose‐derived stem cells (eASCs) in established CIA.

## References

[1] McInnes, I. B. , and G. Schett . 2011 The pathogenesis of rheumatoid arthritis. N Engl. J. Med. 365:2205–2219. 22150039 10.1056/NEJMra1004965

[2] Smolen, J. S. , D. Aletaha , and K. Redlich . 2012 The pathogenesis of rheumatoid arthritis: new insights from old clinical data? Nat. Rev. Rheumatol. 8:235–243. 22410633 10.1038/nrrheum.2012.23

[3] Jiao, Z. , S. Hua , W. Wang , H. Wang , J. Gao , and X. Wang . 2013 Increased circulating myeloid‐derived suppressor cells correlated negatively with Th17 cells in patients with rheumatoid arthritis. Scand. J. Rheumatol. 42:85–90. 23126644 10.3109/03009742.2012.716450

[4] Guo, C. , F. Hu , H. Yi , Z. Feng , C. Li , L. Shi , Y. Li , H. Liu , X. Yu , H. Wang , et al. 2014 Myeloid‐derived suppressor cells have a proinflammatory role in the pathogenesis of autoimmune arthritis. Ann. Rheum. Dis. 1–8. 25371442 10.1136/annrheumdis-2014-205508 PMC4418961

[5] Zhang, H. , S. Wang , Y. Huang , H. Wang , J. Zhao , F. Gaskin , N. Yang , and S. M. Fu . 2015 Myeloid‐derived suppressor cells are proinflammatory and regulate collagen‐induced arthritis through manipulating Th17 cell differentiation. Clin. Immunol. 157:175–186. 25680967 10.1016/j.clim.2015.02.001 PMC4657752

[6] Fujii, W. , E. Ashihara , H. Hirai , H. Nagahara , N. Kajitani , K. Fujioka , K. Murakami , T. Seno , A. Yamamoto , H. Ishino , et al. 2013 Myeloid‐derived suppressor cells play crucial roles in the regulation of mouse collagen‐induced arthritis. J. Immunol. 191:1073–1081. 23804709 10.4049/jimmunol.1203535

[7] Zhang, L. , Z. Zhang , H. Zhang , M. Wu , and Y. Wang . 2014 Myeloid‐derived suppressor cells protect mouse models from autoimmune arthritis via controlling inflammatory response. Inflammation 37:670–677. 24264477 10.1007/s10753-013-9783-z

[8] Wang, W. , Z. Jiao , T. Duan , M. Liu , B. Zhu , Y. Zhang , Q. Xu , R. Wang , Y. Xiong , H. Xu , et al. 2015 Functional characterization of myeloid‐derived suppressor cell subpopulations during the development of experimental arthritis. Eur. J. Immunol. 45:464–473. 25352399 10.1002/eji.201444799

[9] Gabrilovich, D. I. , and S. Nagaraj . 2009 Myeloid‐derived suppressor cells as regulators of the immune system. Nat. Rev. Immunol. 9:162–174. 19197294 10.1038/nri2506 PMC2828349

[10] Zhang, Q. , M. Fujino , J. Xu , and X. K. Li . 2015 The role and potential therapeutic application of myeloid‐derived suppressor cells in allo‐ and autoimmunity. Mediators Inflamm. 2015:1–14. 10.1155/2015/421927 PMC4452474 26078493

[11] Burmester, G. R. , E. Feist , and T. Dorner . 2014 Emerging cell and cytokine targets in rheumatoid arthritis. Nat. Rev. Rheumatol. 10:77–88. 24217582 10.1038/nrrheum.2013.168

[12] Tyndall, A. , and F. A. Houssiau . 2010 Mesenchymal stem cells in the treatment of autoimmune diseases. Ann. Rheum. Dis. 69:1413–1414. 20650875 10.1136/ard.2010.132639

[13] Wang, L. , L. Wang , X. Cong , G. Liu , J. Zhou , B. Bai , Y. Li , W. Bai , M. Li , H. Ji , et al. 2013 Human umbilical cord mesenchymal stem cell therapy for patients with active rheumatoid arthritis: safety and efficacy. Stem Cells Dev. 22:3192–3202. 23941289 10.1089/scd.2013.0023

[14] Papadopoulou, A. , M. Yiangou , E. Athanasiou , N. Zogas , P. Kaloyannidis , I. Batsis , A. Fassas , A. Anagnostopoulos , and E. Yannaki . 2012 Mesenchymal stem cells are conditionally therapeutic in preclinical models of rheumatoid arthritis. Ann. Rheum. Dis. 71:1733–1740. 22586171 10.1136/annrheumdis-2011-200985

[15] Swart, J. F. , and N. M. Wulffraat . 2014 Mesenchymal stromal cells for treatment of arthritis. Best Pract. Res. Clin. Rheumatol. 28:589–603. 25481552 10.1016/j.berh.2014.10.023

[16] Wang, D. , H. Zhang , M. Cao , Y. Tang , J. Liang , X. Feng , H. Wang , B. Hua , B. Liu , and L. Sun . 2011 Efficacy of allogeneic mesenchymal stem cell transplantation in patients with drug‐resistant polymyositis and dermatomyositis. Ann. Rheum. Dis. 70:1285–1288. 21622775 10.1136/ard.2010.141804

[17] Liang, J. , H. Zhang , B. Hua , H. Wang , L. Lu , S. Shi , Y. Hou , X. Zeng , G. S. Gilkeson , and L. Sun . 2010 Allogenic mesenchymal stem cells transplantation in refractory systemic lupus erythematosus: a pilot clinical study. Ann. Rheum. Dis. 69:1423–1429. 20650877 10.1136/ard.2009.123463

[18] Wang, D. , H. Zhang , J. Liang , X. Li , X. Feng , H. Wang , B. Hua , B. Liu , L. Lu , G. S. Gilkeson , et al. 2013 Allogeneic mesenchymal stem cell transplantation in severe and refractory systemic lupus erythematosus: 4 years of experience. Cell Transplant. 22:2267–2277. 24388428 10.3727/096368911X582769c PMC11849135

[19] Nagaishi, K. , Y. Arimura , and M. Fujimiya . 2015 Stem cell therapy for inflammatory bowel disease. J. Gastroenterol. 50:280–286. 25618180 10.1007/s00535-015-1040-9

[20] English, K. , A. French , and K. J. Wood . 2010 Mesenchymal stromal cells: facilitators of successful transplantation? Cell Stem Cell 7:431–442. 20887949 10.1016/j.stem.2010.09.009

[21] Glenn, J. D. , and K. A. Whartenby . 2014 Mesenchymal stem cells: Emerging mechanisms of immunomodulation and therapy. World J. Stem Cells 6:526–539. 25426250 10.4252/wjsc.v6.i5.526 PMC4178253

[22] Swart, J. F. , S. de Roock , F. M. Hofhuis , H. Rozemuller , T. van den Broek , P. Moerer , F. Broere , F. van Wijk , W. Kuis , B. J. Prakken , et al. 2015 Mesenchymal stem cell therapy in proteoglycan induced arthritis. Ann. Rheum. Dis. 74:769–777. 24395558 10.1136/annrheumdis-2013-204147

[23] Gonzalez, M. A. , E. Gonzalez‐Rey , L. Rico , D. Buscher , and M. Delgado . 2009 Treatment of experimental arthritis by inducing immune tolerance with human adipose‐derived mesenchymal stem cells. Arthritis Rheum. 60:1006–1019. 19333946 10.1002/art.24405

[24] Gonzalez‐Rey, E. , M. A. Gonzalez , N. Varela , F. O'Valle , P. Hernandez‐Cortes , L. Rico , D. Buscher , and M. Delgado . 2010 Human adipose‐derived mesenchymal stem cells reduce inflammatory and T cell responses and induce regulatory T cells in vitro in rheumatoid arthritis. Ann. Rheum. Dis. 69:241–248. 19124525 10.1136/ard.2008.101881

[25] Lopez‐Santalla, M. , P. Mancheno‐Corvo , R. Menta , J. Lopez‐Belmonte , O. DelaRosa , J. A. Bueren , W. Dalemans , E. Lombardo , and M. Garín . 2015 Human adipose‐derived mesenchymal stem cells modulate experimental autoimmune arthritis by modifying early adaptive T cell responses. Stem Cells 33:3493–3503. 26205964 10.1002/stem.2113

[26] Soleymaninejadian, E. , K. Pramanik , and E. Samadian . 2012 Immunomodulatory properties of mesenchymal stem cells: cytokines and factors. Am. J. Reprod. Immunol. 67:1–8. 21951555 10.1111/j.1600-0897.2011.01069.x

[27] Abomaray, F. M. , M. A. Al Jumah , B. Kalionis , A. S. AlAskar , S. Al Harthy , D. Jawdat , A. Al Khaldi , A. Alkushi , B. A. Knawy , and M. H. Abumaree . 2014 Human chorionic villous mesenchymal stem cells modify the functions of human dendritic cells, and induce an anti‐inflammatory phenotype in CD1+ dendritic cells. Stem Cell Rev. 11(3):423–441. 25287760 10.1007/s12015-014-9562-8

[28] Gonzalez‐Rey, E. , P. Anderson , M. A. Gonzalez , L. Rico , D. Buscher , and M. Delgado . 2009 Human adult stem cells derived from adipose tissue protect against experimental colitis and sepsis. Gut 58:929–939. 19136511 10.1136/gut.2008.168534

[29] Nemeth, K. , A. Leelahavanichkul , P. S. Yuen , B. Mayer , A. Parmelee , K. Doi , P. G. Robey , K. Leelahavanichkul , B. H. Koller , J. M. Brown , et al. 2009 Bone marrow stromal cells attenuate sepsis via prostaglandin E(2)‐dependent reprogramming of host macrophages to increase their interleukin‐10 production. Nat. Med. 15:42–49. 19098906 10.1038/nm.1905 PMC2706487

[30] Chou, H. S. , C. C. Hsieh , H. R. Yang , L. Wang , Y. Arakawa , K. Brown , Q. Wu , F. Lin , M. Peters , J. J. Fung , et al. 2011 Hepatic stellate cells regulate immune response by way of induction of myeloid suppressor cells in mice. Hepatology 53:1007–1019. 21374665 10.1002/hep.24162 PMC3079329

[31] Melief, S. M. , S. B. Geutskens , W. E. Fibbe , and H. Roelofs . 2013 Multipotent stromal cells skew monocytes towards an anti‐inflammatory function: the link with key immunoregulatory molecules. Haematologica 98:e121–122. 24006414 10.3324/haematol.2013.093864 PMC3762117

[32] Obermajer, N. , F. C. Popp , Y. Soeder , J. Haarer , E. K. Geissler , H. J. Schlitt , and M. H. Dahlke . 2014 Conversion of Th17 into IL‐17A(neg) regulatory T cells: a novel mechanism in prolonged allograft survival promoted by mesenchymal stem cell‐supported minimized immunosuppressive therapy. J. Immunol. 193:4988–4999. 25305313 10.4049/jimmunol.1401776

[33] Lee, H. J. , J. H. Ko , H. J. Jeong , A. Y. Ko , M. K. Kim , W. R. Wee , S. O. Yoon , and J. Y. Oh . 2015 Mesenchymal stem/stromal cells protect against autoimmunity via CCL2‐dependent recruitment of myeloid‐derived suppressor cells. J. Immunol. 194:3634–3645. 25769927 10.4049/jimmunol.1402139

[34] Li, Y. , Z. Tu , S. Qian , J. J. Fung , S. D. Markowitz , L. L. Kusner , H. J. Kaminski , L. Lu , and F. Lin . 2014 Myeloid‐derived suppressor cells as a potential therapy for experimental autoimmune myasthenia gravis. J. Immunol. 193:2127–2134. 25057008 10.4049/jimmunol.1400857 PMC4784709

[35] Anderson, P. , L. Souza‐Moreira , M. Morell , M. Caro , F. O'Valle , E. Gonzalez‐Rey , and M. Delgado . 2013 Adipose‐derived mesenchymal stromal cells induce immunomodulatory macrophages which protect from experimental colitis and sepsis. Gut 62:1131–1141. 22637701 10.1136/gutjnl-2012-302152

[36] Luz‐Crawford, P. , G. Tejedor , A. L. Mausset‐Bonnefont , E. Beaulieu , E. F. Morand , C. Jorgensen , D. Noel , and F. Djouad . 2015 Gilz governs the therapeutic potential of mesenchymal stem cells by inducing a switch from pathogenic to regulatory Th17 cells. Arthritis Rheumatol 67(6):1514–1524. 25708718 10.1002/art.39069

[37] Zhou, B. , J. Yuan , Y. Zhou , M. Ghawji, Jr. , Y. P. Deng , A. J. Lee , A. J. Lee , U. Nair , A. H. Kang , D. D. Brand , et al. 2011 Administering human adipose‐derived mesenchymal stem cells to prevent and treat experimental arthritis. Clin. Immunol. 141:328–337. 21944669 10.1016/j.clim.2011.08.014

[38] Augello, A. , R. Tasso , S. M. Negrini , R. Cancedda , and G. Pennesi . 2007 Cell therapy using allogeneic bone marrow mesenchymal stem cells prevents tissue damage in collagen‐induced arthritis. Arthritis Rheum. 56:1175–1186. 17393437 10.1002/art.22511

[39] Zigmond, E. , C. Varol , J. Farache , E. Elmaliah , A. T. Satpathy , G. Friedlander , M. Mack , N. Shpigel , I. G. Boneca , K. M. Murphy , et al. 2012 Ly6C hi monocytes in the inflamed colon give rise to proinflammatory effector cells and migratory antigen‐presenting cells. Immunity 37:1076–1090. 23219392 10.1016/j.immuni.2012.08.026

[40] Tamoutounour, S. , M. Guilliams , F. Montanana Sanchis , H. Liu , D. Terhorst , C. Malosse , E. Pollet , L. Ardouin , H. Luche , C. Sanchez , et al. 2013 Origins and functional specialization of macrophages and of conventional and monocyte‐derived dendritic cells in mouse skin. Immunity 39:925–938. 24184057 10.1016/j.immuni.2013.10.004

[41] Smolen, J. S. , and G. Steiner . 2003 Therapeutic strategies for rheumatoid arthritis. Nat. Rev. Drug Discov. 2:473–488. 12776222 10.1038/nrd1109

[42] Cook, A. D. , J. Pobjoy , S. Sarros , S. Steidl , M. Durr , D. C. Lacey , and J. A. Hamilton . 2013 Granulocyte‐macrophage colony‐stimulating factor is a key mediator in inflammatory and arthritic pain. Ann. Rheum. Dis. 72:265–270. 22833372 10.1136/annrheumdis-2012-201703

[43] Burmester, G. R. , M. E. Weinblatt , I. B. McInnes , D. Porter , O. Barbarash , M. Vatutin , I. Szombati , E. Esfandiari , M. A. Sleeman , C. D. Kane , et al. 2013 Efficacy and safety of mavrilimumab in subjects with rheumatoid arthritis. Ann. Rheum. Dis. 72:1445–1452. 23234647 10.1136/annrheumdis-2012-202450 PMC3756523

[44] Behrens, F. , P. P. Tak , M. Ostergaard , R. Stoilov , P. Wiland , T. W. Huizinga , V. Y. Berenfus , S. Vladeva , J. Rech , A. Rubbert‐Roth , et al. 2014 MOR103, a human monoclonal antibody to granulocyte‐macrophage colony‐stimulating factor, in the treatment of patients with moderate rheumatoid arthritis: results of a phase Ib/IIa randomised, double‐blind, placebo‐controlled, dose‐escalation trial. Ann. Rheum. Dis. 74(6):1058–1064. 24534756 10.1136/annrheumdis-2013-204816 PMC4431325

[45] Greven, D. E. , E. S. Cohen , D. M. Gerlag , J. Campbell , J. Woods , N. Davis , A. van Nieuwenhuijze , A. Lewis , S. Heasmen , M. McCourt , et al. 2014 Preclinical characterisation of the GM‐CSF receptor as a therapeutic target in rheumatoid arthritis. Ann. Rheum. Dis. 1–7. 24936585 10.1136/annrheumdis-2014-205234 PMC4602263

[46] Toupet, K. , M. Maumus , P. Luz‐Crawford , E. Lombardo , J. Lopez‐Belmonte , P. van Lent , M. I. Garin , W. van den Berg , W. Dalemans , C. Jorgensen , et al. 2015 Survival and biodistribution of xenogenic adipose mesenchymal stem cells is not affected by the degree of inflammation in arthritis. PLoS One 10:e0114962. 25559623 10.1371/journal.pone.0114962 PMC4283953

[47] Eggenhofer, E. , F. Luk , M. H. Dahlke , and M. J. Hoogduijn . 2014 The life and fate of mesenchymal stem cells. Front Immunol. 19:148. 24904568 10.3389/fimmu.2014.00148 PMC4032901

[48] Melief, S. M. , E. Schrama , M. H. Brugman , M. M. Tiemessen , M. J. Hoogduijn , W. E. Fibbe , and H. Roelofs . 2013 Multipotent stromal cells induce human regulatory T cells through a novel pathway involving skewing of monocytes toward anti‐inflammatory macrophages. Stem Cells 31:1980–1991. 23712682 10.1002/stem.1432

[49] Hoechst, B. , J. Gamrekelashvili , M. P. Manns , T. F. Greten , and F. Korangy . 2011 Plasticity of human Th17 cells and iTregs is orchestrated by different subsets of myeloid cells. Blood 117:6532–6541. 21493801 10.1182/blood-2010-11-317321

[50] Beyth, S. , Z. Borovsky , D. Mevorach , M. Liebergall , Z. Gazit , H. Aslan , E. Galun , and J. Rachmilewitz . 2005 Human mesenchymal stem cells alter antigen‐presenting cell maturation and induce T‐cell unresponsiveness. Blood 105:2214–2219. 15514012 10.1182/blood-2004-07-2921

[51] Assis, A. C. , J. L. Carvalho , B. A. Jacoby , R. L. Ferreira , P. Castanheira , S. O. Diniz , V. N. Cardoso , A. M. Goes , and A. J. Ferreira . 2010 Time‐dependent migration of systemically delivered bone marrow mesenchymal stem cells to the infarcted heart. Cell Transplant. 19:219–230. 19906330 10.3727/096368909X479677

[52] Eggenhofer, E. , V. Benseler , A. Kroemer , F. C. Popp , E. K. Geissler , H. J. Schlitt , C. C. Baan , M. H. Dahlke , and M. J. Hoogduijn . 2012 Mesenchymal stem cells are short‐lived and do not migrate beyond the lungs after intravenous infusion. Front Immunol. 26:297. 23056000 10.3389/fimmu.2012.00297 PMC3458305

[53] Yen, B. L. , M. L. Yen , P. J. Hsu , K. J. Liu , C. J. Wang , C. H. Bai , and H. K. Sytwu . 2013 Multipotent human mesenchymal stromal cells mediate expansion of myeloid‐derived suppressor cells via hepatocyte growth factor/c‐met and STAT3. Stem Cell Rep. 1:139–151. 10.1016/j.stemcr.2013.06.006 PMC3757753 24052949

[54] Campioni, D. , D. Bortolotti , O. R. Baricordi , and R. Rizzo . 2013 Multipotent stromal cells skew monocytes towards an anti‐inflammatory function: a role for HLA‐G molecules. Haematologica 98:e114. 24006410 10.3324/haematol.2013.090092 PMC3762113

[55] van Baarsen, L. G. , M. J. de Hair , T. H. Ramwadhdoebe , I. J. Zijlstra , M. Maas , D. M. Gerlag , and P. P. Tak . 2013 The cellular composition of lymph nodes in the earliest phase of inflammatory arthritis. Ann. Rheum. Dis. 72:1420–1424. 23661491 10.1136/annrheumdis-2012-202990 PMC3711496

[56] Park, M. J. , H. S. Park , M. L. Cho , H. J. Oh , Y. G. Cho , S. Y. Min , B. H. Chung , J. W. Lee , H. Y. Kim , and S. G. Cho . 2011 Transforming growth factor beta‐transduced mesenchymal stem cells ameliorate experimental autoimmune arthritis through reciprocal regulation of Treg/Th17 cells and osteoclastogenesis. Arthritis Rheum. 63:1668–1680. 21384335 10.1002/art.30326

