# Supplementary figures and images for: Adipose‐derived mesenchymal stromal cells modulate experimental autoimmune arthritis by inducing an early regulatory innate cell signature
Source: Immun Inflamm Dis. 2016 Apr 4;4(2):213–24. doi: 10.1002/iid3.106 (PMC4879467; doi:10.1002/iid3.106)

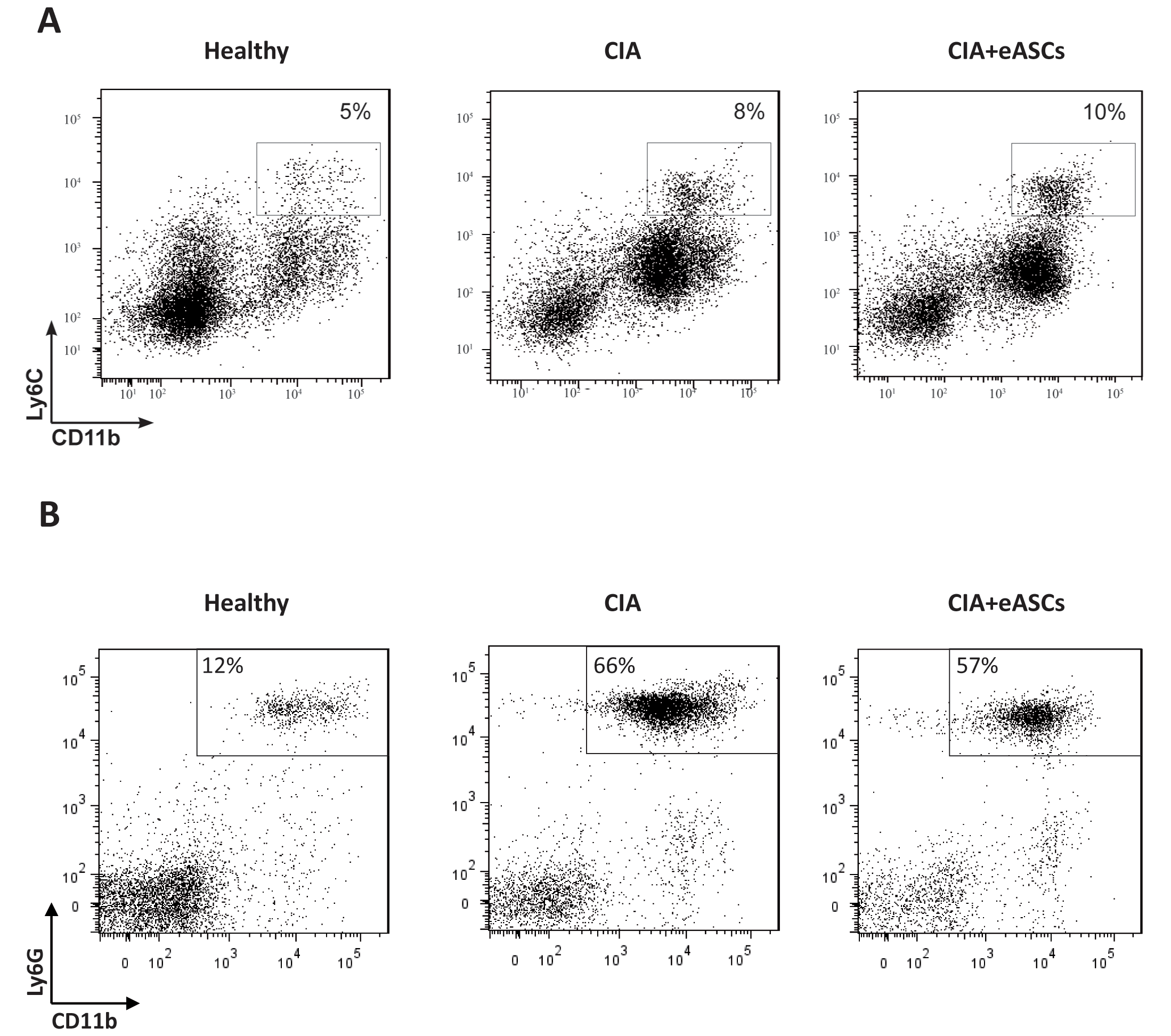

Supplement: Supplementary file 1 — Figure S1. Quantification of Ly6C+CD11b+ and Ly6G+CD11b+ myeloid cell populations in peripheral blood by flow cytometry. [file IID3-4-213-s001.tif]

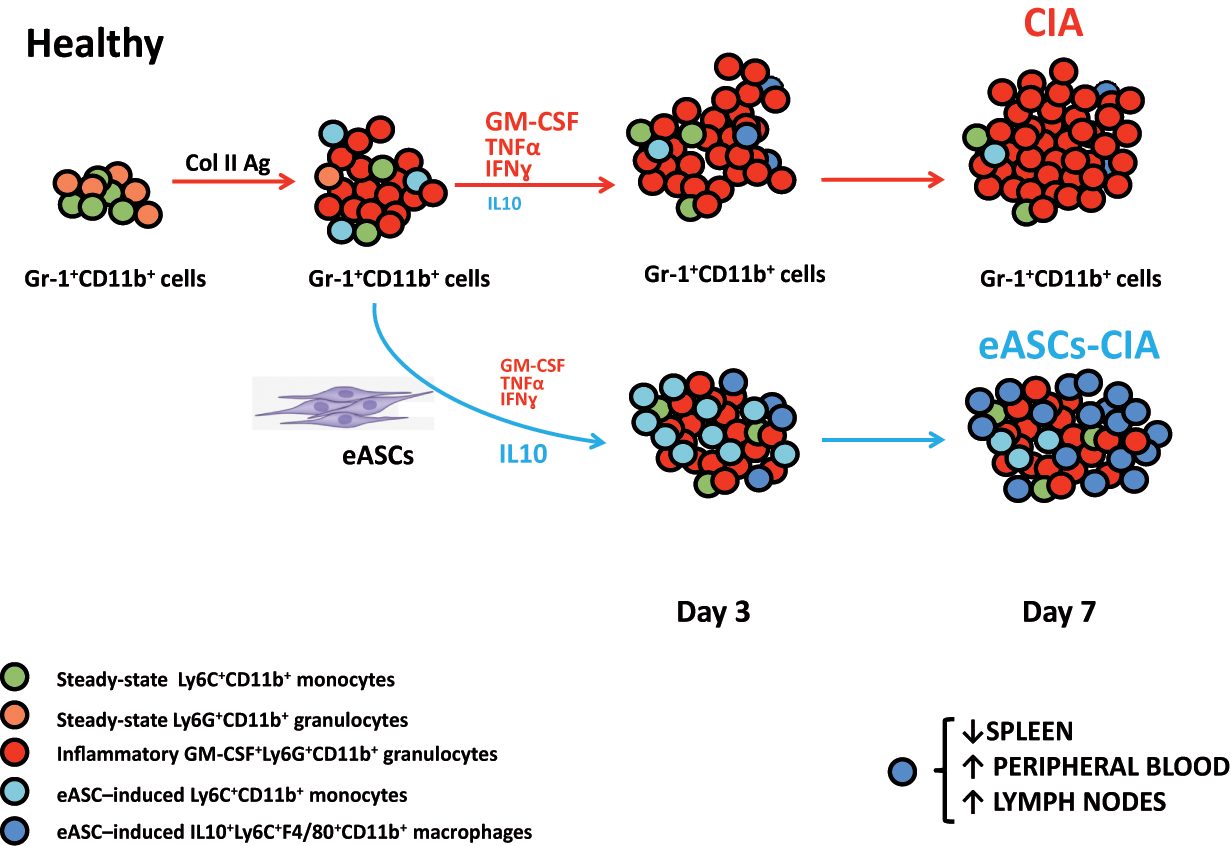

Supplement: Supplementary file 2 — Figure S2. Schematic model for early innate responses induced by the expanded adipose‐derived stem cells (eASCs) in established CIA. [file IID3-4-213-s002.tif]
